# Towards Terawatt Sub-Cycle Long-Wave Infrared Pulses via Chirped Optical Parametric Amplification and Indirect Pulse Shaping

**DOI:** 10.1038/srep45794

**Published:** 2017-04-03

**Authors:** Yanchun Yin, Andrew Chew, Xiaoming Ren, Jie Li, Yang Wang, Yi Wu, Zenghu Chang

**Affiliations:** 1 Institute for the Frontier of Attosecond Science and Technology, CREOL and Department of Physics, University of Central Florida, Orlando, Florida, 32816, USA

## Abstract

We present an approach for both efficient generation and amplification of 4–12 *μ*m pulses by tailoring the phase matching of the nonlinear crystal Zinc Germanium Phosphide (ZGP) in a narrowband-pumped optical parametric chirped pulse amplifier (OPCPA) and a broadband-pumped dual-chirped optical parametric amplifier (DC-OPA), respectively. Preliminary experimental results are obtained for generating 1.8–4.2 *μ*m super broadband spectra, which can be used to seed both the signal of the OPCPA and the pump of the DC-OPA. The theoretical pump-to-idler conversion efficiency reaches 27% in the DC-OPA pumped by a chirped broadband Cr^2+^:ZnSe/ZnS laser, enabling the generation of  Terawatt-level 4–12 *μ*m pulses with an available large-aperture ZGP. Furthermore, the 4–12 *μ*m idler pulses can be compressed to sub-cycle pulses by compensating the tailored positive chirp of the idler pulses using the bulk compressor NaCl, and by indirectly controlling the higher-order idler phase through tuning the signal (2.4–4.0 *μ*m) phase with a commercially available acousto-optic programmable dispersive filter (AOPDF). A similar approach is also described for generating high-energy 4–12 *μ*m sub-cycle pulses via OPCPA pumped by a 2 *μ*m Ho:YLF laser.

So far, isolated attosecond pulses as short as 67 as have been generated via high harmonic generation (HHG) driven by few-cycle near infrared pulses at ~800 nm^1^. Because the single atom cutoff photon energy of HHG is proportional to wavelength squared, long-wavelength laser sources are needed to extend the cutoff^2^,^3^. Recently, two-cycle mJ-level driving lasers around 1.7 *μ*m has been used to generate high-flux soft X-ray pulses in the water-window region (280 to 530 eV)^4^,^5^, supporting 10 as transform limited pulses. To significantly increase both center photon energy and bandwidth of high harmonics for generating shorter attosecond or even zeptosecond X-ray pulses, the development of high-energy few-cycle pulses further into the mid-infrared (mid-IR) region is in demand. Beyond attosecond science, the high-energy few-cycle long-wavelength pulses are ideal tools for studying incoherent hard X-ray generation^6^, acceleration of electrons in dielectric structures and plasma^7^,^8^,^9^, breakdown of dipole approximation^10^ and filamentation in air. In addition, the high-repetition-rate few-cycle long-wavelength pulses would benefit the study of rotational and vibrational dynamics of molecules^11^, time-resolved imaging of molecular structures^12^, and strong-field science in plasmonic systems^13^.

High-energy few-cycle infrared pulses are primarily enabled by optical parametric chirped pulse amplification (OPCPA) pumped by few-picosecond lasers, which allows a trade-off between the gain bandwidth and the damage threshold of nonlinear crystals^14^,^15^. Among a number of few-cycle IR OPCPA sources that have been reported^16^,^17^,^18^,^19^,^20^,^21^,^22^, only a few of them have yielded mJ-level, carrier-envelope-phase (CEP)-stable, and near-octave pulses (two cycles and below) at-1.2–2.2 *μ*m^21^,^22^ and 1.6–2.7 *μ*m^18^. We have previously experimentally demonstrated 3 mJ, CEP-stable two-cycle pulses from 1.2 *μ*m to 2.2 *μ*m with the highest 18% pump-to-signal conversion efficiency^22^ and numerically demonstrated for the first time a robust approach for generation of mJ-level, CEP-stable, two-cycle pulses from 2.4 *μ*m to 4.0 *μ*m^23^. Recently, rapid progress has been made on the generation of short-pulse mid-IR laser sources around 5 *μ*m or above^24^,^25^,^26^. However, the compression of such pulses down to the transform limit has remained a great challenge due to lack of higher-order phase control over broadband mid-IR.

In this paper, we present a design that enables not only generating, for the first time to the best of our knowledge, mJ-level, CEP-stable 4–12 *μ*m pulses through dual-chirped optical parametric amplification (DC-OPA)^22^,^23^,^27^,^28^,^29^,^30^, but also compressing such pulses to sub-cycle duration through indirect pulse shaping. We have experimentally generated a *μJ*-level super broadband spectrum, which covers 1.8–4.2 *μ*m generated by difference frequency generation (DFG) in a BIBO crystal. On one hand, the spectrum can be used to seed the signal for generating 4–12 *μ*m pulses in an OPCPA pumped by a 2 *μ*m narrowband laser source; on the other hand, the 1.8–4.2 *μ*m pulses can be used to seed a Cr^2+^:ZnSe/ZnS chirped pulse amplifier (CPA), which is used for pumping the amplification stage of the 4–12 *μ*m DC-OPA. The 4–12 *μ*m optical parametric bandwidth is achieved by tailoring the phase matching of ZGP. The second-order phase of high-energy 4–12 *μ*m pulses can be compensated by using NaCl, which has a very small n_2_ and a high damage threshold; the higher-order phase can be compensated indirectly by controlling the signal phase with a commercially available acousto-optic programmable dispersive filter (AOPDF).

## Results

**Figure 1:**
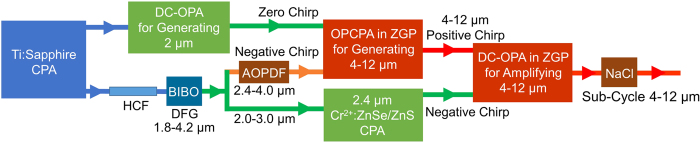
Proof-of-principle schematic setup for generating, amplifying, and compressing 4–12 *μ*m pulses.

A proof-of-principle schematic setup shown in Fig. 1, which will be analyzed in detail, is used to facilitate all discussions in this paper. In this section, we first present the experimental generation of a *μJ*-level super broadband spectrum, which covers 1.8–4.2 *μ*m generated by DFG in a BIBO crystal, followed by discussions on how to tailor the phase matching of ZGP in order to achieve 4–12 *μ*m parametric bandwidth and on indirect pulse shaping toward sub-cycle pulses. Numerical simulations on generation in an OPCPA and amplification in either a DC-OPA or an OPCPA of 4–12 *μ*m pulses are shown in the latter part of this section.

### Generation of super broadband spectra via DFG

We first present the results for generating broadband seed pulses through intra-pulse DFG. DFG is commonly used to generate the seed source due to its broad optical parametric bandwidth and passive-stable CEP^16^,^18^,^19^,^20^,^31^. Here we used spectrally broadened Ti:Sapphire pulses as the driving source for DFG. Nanojoule-level Ti:Sapphire oscillator pulses (730–830 nm) were stretched to 360 ps in an Offner-type stretcher, boosted to 4 mJ with a home-made 14-pass amplifier, and compressed to 30 fs with 3.5 mJ pulse energy. Part of the energy, 2.2 mJ, was focused into a 0.5-mm-diameter hollow core fiber (HCF) filled with 30 psi neon for white light generation (WLG). The WLG pulses from the HCF were compressed to below 7 fs. Finally 0.75 mJ, <7 fs pulses were obtained for driving DFG.

**Figure 2:**
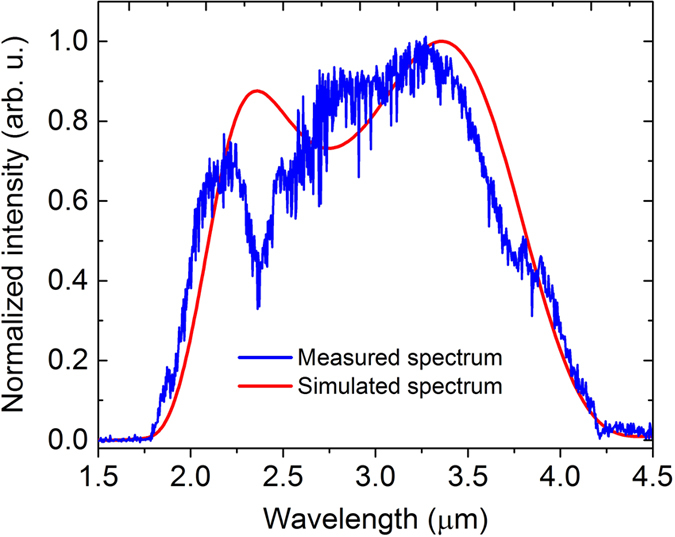
DFG in BIBO for generating 1.8–4.2 *μ*m broadband seed pulses.

Guided by numerical simulation, we experimentally demonstrated that BIBO could generate more than one octave spectrum. Figure 2 shows the 1.8–4.2 *μ*m DFG with 15 *μ*J energy experimentally achieved in a 0.4 *μ*m BIBO crystal, which falls under the gain spectrum of Cr^2+^:ZnSe/ZnS crystals^32^ and could be amplified in a Cr^2+^:ZnSe/ZnS CPA laser system^33^ that serves as the pump for the amplification stage of the 4–12 *μ*m DC-OPA. Meanwhile, the 2.4–4.0 *μ*m portion of the spectrum can be used as the signal for seeding the generation stage of the 4–12 *μ*m OPCPA. This is the first time, to the best of our knowledge, that such broadband pulses have been generated in BIBO. Moreover, the 15 *μ*J energy is sufficiently high enough for studying high-harmonic generation in solids^34^.

### Phase matching tailoring and indirect pulse shaping

In an OPA, the phase of the third output wave is determined by the phases of the two input waves:


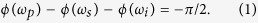


Hence, it is feasible to indirectly control the phase of the third wave by controlling the phase of either of the two input waves. In a DC-OPA, if both the pump and the signal are linearly chirped, *ω*_*p,s*_(*t*) = *ω*_*p*0,*s*0_ + *β*_*p,s*_*t*, in which *ω*_0_ is the central angular frequency and *β* is the chirp (the subscripts *p* and *s* refer to the pump and signal, respectively). By conservation of energy, the angular frequency of the idler is thus


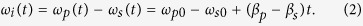


**Figure 3:**
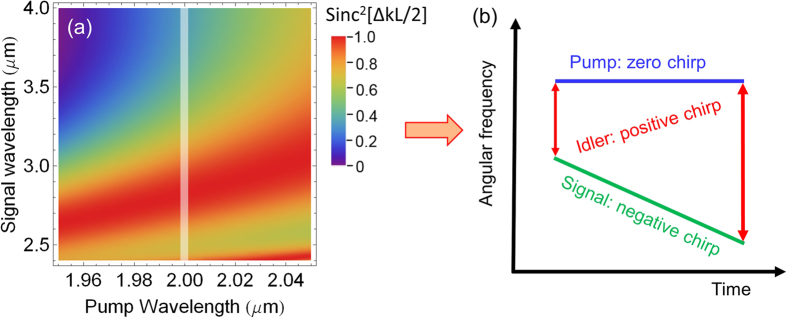
(**a**) The calculated phase matching efficiency (Sinc^2^[ΔkL/2]) as a function of pump and signal wavelengths for a 0.6 mm Type I (phase matching angle: 54°) ZGP crystal. Δk is the propagation constant difference and L is the ZGP crystal length. The white shaded line qualitatively shows the utilized phase matching region in the 4–12 *μ*m OPCPA. (**b**) Chirp relationship among the pump, signal, and idler.

Hence, the chirp of the generated idler pulse is equal to the difference between the chirps of the pump and signal pulses. Chirp management in a DC-OPA has been analyzed and experimentally utilized to generate both few-cycle IR laser pulses^22^,^23^,^28^,^29^,^30^ and tunable narrowband mid-IR laser pulses^27^. Here we use chirp management combined with phase-matching tailoring to not only generate the 4–12 *μ*m broadband laser pulses, but also compress such broadband pulses to sub-cycle pulse duration via indirect pulse shaping. ZnGeP_2_ (ZGP) crystal is chosen as the nonlinear crystal as it has a high second-order nonlinearity (~80 pm/V), wide transparency, and broad parametric bandwidth. There are several considerations on phase-matching tailoring and indirect pulse shaping in order to generate and compress the 4–12 *μ*m idler pulses:

1. Bandwidth. Phase-matching tailoring is a powerful tool for generating a desired output bandwidth using currently available input laser bandwidth. It can be qualitatively seen from the shaded white line in Fig. 3(a) that a pump pulse (2 *μ*m) and a signal pulse (2.4–4.0 *μ*m) can generate a 4–12 *μ*m idler pulse. The pump and signal bandwidth are within the bandwidth generated by DFG in BIBO.
2. Dispersion. For few-cycle pulse compression, a common and robust approach is to use a bulk material to control the second-order phase (chirp) and use an AOPDF to control the higher-order phase. Although there is AOPDF that can work beyond 4 *μ*m^35^,^36^, the rather low efficiency makes such AOPDF only applicable to narrowband mid-IR pulses. Since the chirp of the idler pulse is determined by the chirp of the pump pulse and the signal pulse, here we propose to compensate the higher-order phase of the idler pulse indirectly by controlling the higher-order phase of the signal pulse with an AOPDF that can work efficiently in the spectral range covering 2.4–4.0 *μ*m. To compensate a larger portion of chirp of the 4–12 *μ*m idler pulse, the search for an appropriate bulk material is also critical. While there are some materials that have good transparency in 4–12 *μ*m, most of them are not appropriate for compression of high-energy few-cycle pulses. For example, Ge has a small bandgap and a high n_2_ and ZnSe/ZnS has a large second-order nonlinearity. Here we found that NaCl crystal, which has a large bandgap (9 eV) and a small n_2_, would be a great choice for a bulk compressor and is commercially available in large size. As NaCl has a negative chirp in 4–12 *μ*m, the chirp of the 4–12 *μ*m idler pulse needs to be positive before compression, which further determines that the signal chirp needs to be negative, as shown in Fig. 3(b).
3. Spectral resolution of phase control for indirect pulse shaping. As the signal bandwidth (2.4–4.0 *μ*m) is relatively narrow compared to the idler bandwidth (4–12 *μ*m), a finite signal spectral resolution will translate to an idler spectral resolution that is 5 times lower. For example, the best resolution of an AOPDF around the signal bandwidth is around 1 to 2 nm, which translates to a resolution of 5–10 nm in the idler spectral region. There are still 800 to 1600 resolution points in 4–12 *μ*m, which are well enough for compression using an AOPDF as it gives continuous phase control, unlike spatial light modulators that only control phase at discrete wavelength points.

### Simulation of 4–12 *μ*m idler pulse generation

Due to the many advantages mentioned in the last section, ZGP has been used in both OPA and OPCPA pumped around 2 *μ*m to generate short MIR laser pulses. For example, an OPA in ZGP generated 53 *μ*J, 700 fs laser pulses at 5 *μ*m when pumped by another OPA at around 2 *μ*m^24^; an OPCPA in ZGP generated 100 *μ*J, 69 fs (calculated transform limit) laser pulses at 5.3 *μ*m when pumped by a Ho:YAG CPA at 2.09 *μ*m^26^; another OPCPA in ZGP generated 200 *μ*J, 360 fs laser pulses at 7 *μ*m when pumped by a Ho:YLF CPA at 2 *μ*m^25^. Here we simulate the OPCPA discussed in the last section to generate 4–12 *μ*m idler pulses. A one-dimensional three-wave mixing numerical model has been developed by modifying the previous one^37^. All three waves are assumed to be plane waves. This model can be applied more accurately to situations where a flat top beam profile and a flat top pulse shape are used.

**Figure 4:**
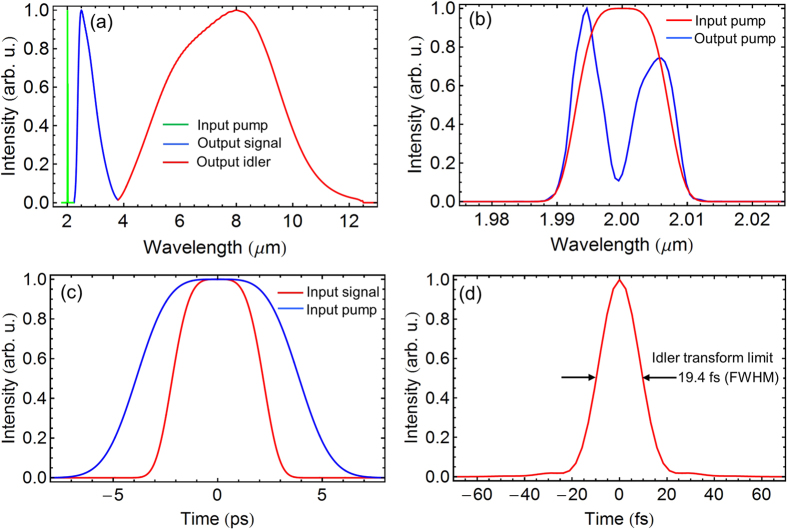
Simulation results for generating 4–12 *μ*m idler pulses in a 0.4-mm-ZGP OPCPA pumped by a 2 *μ*m narrowband laser: (**a**) input pump spectrum (green), output signal spectrum (blue), and generated idler spectrum (red); (**b**) input (red) and output (blue) pump spectra; (**c**) input chirped pump (blue) and signal (red) pulse shapes; (**d**) calculated transform-limited pulse duration (FWHM) of the generated idler: 19.4 fs.

In the first simulation, the phase matching tailored in Fig. 3(a) is used to demonstrate the generation of the 4–12 *μ*m idler. The 2.4–4.0 *μ*m signal pulse, which can be generated in BIBO using DFG, is negatively stretched to 6.0 ps, while the 2.0 *μ*m pump pulse has a pulse duration (FWHM) of 7.8 ps, as shown in Fig. 4(c). The input signal energy is assumed to be 1 *μ*J, which is realistic according to the BIBO DFG experiment. The input pump energy is 100 *μ*J. The input signal and pump intensities are 0.44 GW/cm^2^ and 20 GW/cm^2^, respectively, which are realistic since ZGP has a high damage threshold and can withstand 20 GW/cm^2^ intensity at 2 *μ*m and 10 ps pulse duration^25^. The pump-to-signal pulse duration ratio is chosen to optimize the parametric gain bandwidth without losing significant conversion efficiency. The ZGP crystal has a Type I phase-matching angle of 54°. The optimized crystal length is 0.4 mm, which can give a decent conversion efficiency without reduction of the 4–12 *μ*m parametric gain bandwidth. The simulation results are shown in Fig. 4. As shown in Fig. 4(a), the amplified idler spectrum spans from 4 *μ*m to 12 *μ*m, supporting a 19.4 fs (FWHM) transform-limited sub-cycle pulse, as can be seen from Fig. 4(d). The pump-to-idler conversion efficiency reaches 11.8% with a signal gain of 23.4, which can give an output idler energy more than 10 *μ*J.

**Figure 5:**
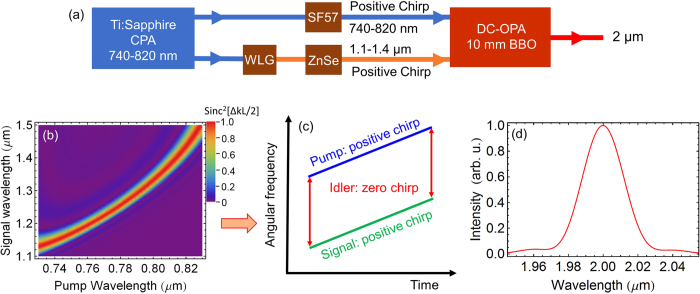
(**a**) Proof-of-principle schematic setup for generating the narrowband 2 *μ*m pump in a DC-OPA. WLG: white light generation. (**b**) The calculated phase matching efficiency (Sinc^2^[ΔkL/2]) as a function of pump and signal wavelengths for a 10 mm Type I (phase matching angle: 20°) BBO crystal. Δk is the propagation constant difference phase and L is the BBO crystal length. (**c**) Chirp management among the pump, signal, and idler. (**c**) Generated idler spectrum centered at 2 *μ*m.

It is worth mentioning the synchronization between the pump and the signal. While the 2 *μ*m pump source can be generated with a Ho:YLF amplifier seeded with the BIBO DFG, the narrowband 2 *μ*m would have very low energy, which needs to be amplified with a regenerative Ho:YLF amplifier. However, the long optical path (several hundred meters) of the regenerative amplifier would make it rather difficult to synchronize the pump and the signal. Here, a DC-OPA is proposed instead to generate the narrowband 2 *μ*m pump, as shown in Fig. 5(a). Broadband spectrum around 2 *μ*m has been demonstrated using OPA in BBO^38^. In order to generate the narrowband 2 *μ*m spectrum, the DC-OPA is designed using the phase matching shown in Fig. 5(b). The key principle, illustrated in Fig. 5(c), is that when the pump and signal have an equal amount of chip, the generated idler would have a zero chirp and thus have narrow bandwidth. The idler bandwidth and center wavelength can be easily tuned by changing the chirps of the pump and signal and the delay between them, respectively. One example for the generated spectrum is shown in Fig. 5(d). In the simulation, the pump pulse is positively chirped using a 13.5-mm thick SF57, while the signal pulse is positively chirped using a 6.2-mm thick ZnSe. The center wavelengths of the pump (790 nm) and the signal (1298 nm) have an equal group delay dispersion of 3016 fs^2^. The pump and signal intensities are 10.5 GW/cm^2^ and 1 kW/cm^2^, respectively. The BBO thickness is set to be 10 mm with a Type I phase-matching angle of 20°. The pump-to-idler efficiency reaches 22.7% with a signal gain of 4 × 10^6^, which can give more than 200 *μ*J energy around 2.0 *μ*m with a 1 mJ Ti:Sapphire pump.

### Simulation of 4–12 *μ*m idler pulse amplification

**Figure 6:**
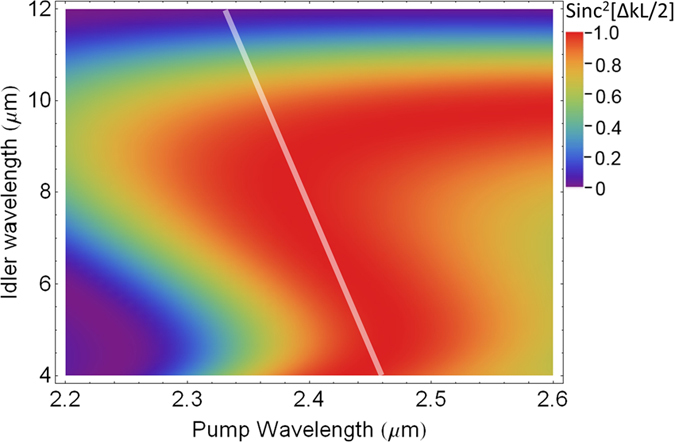
The calculated phase matching efficiency (Sinc^2^[ΔkL/2]) as a function of pump and signal wavelengths for a 1.5 mm Type I (phase matching angle: 48.5°) ZGP crystal. Δk is the propagation constant difference and L is the ZGP crystal length. The white shaded line qualitatively shows the utilized phase matching region for amplifying 4–12 *μ*m idler pulses in the DC-OPA pumped by a 2.4 *μ*m Cr^2+^:ZnSe/ZnS CPA.

Recently, the rapid development of Cr^2+^:ZnSe/ZnS CPA has opened up the opportunity to pump ZGP at a longer wavelength^32^,^33^. Ideally, ZGP should be pumped at around 2.5 *μ*m in order to achieve the broadest parametric bandwidth. Unlike the pump wavelength required for generating 4–12 *μ*m idler pulses in ZGP, which is predetermined by the available signal wavelength, the pump wavelength required for amplifying 4–12 *μ*m idler pulses is quite flexible, which can be tailored to meet a convenient gain bandwidth of Cr^2+^:ZnSe/ZnS CPA. The required pump center wavelength can be tailored by changing the phase-matching angle. One example of such phase-matching tailoring is shown by the shaded line in Fig. 6, which shows that the chirp signs of the pump and the idler have to be opposite. Since the chirp of the idler, which has been predetermined inside the OPCPA for generating the idler, is positive, the chirp of the pump has to be negative.

**Figure 7:**
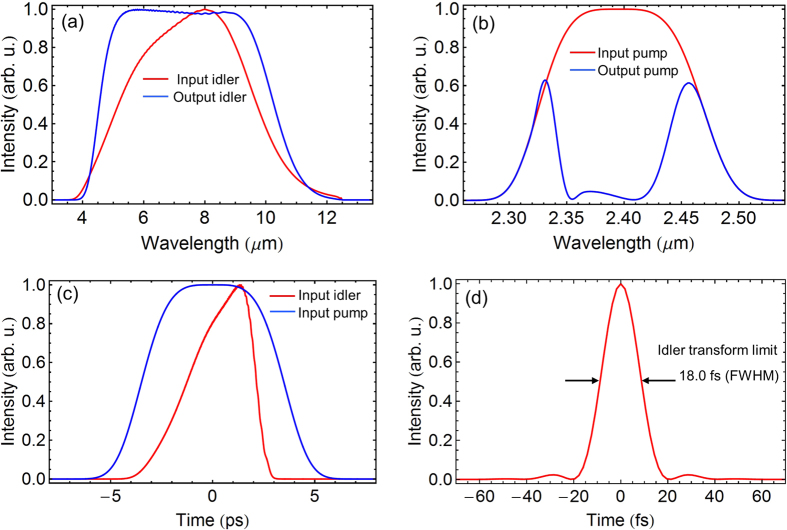
Simulation results for amplifying 4–12 *μ*m idler pulses in a 0.76-mm-ZGP DC-OPA pumped by a 2.4 *μ*m Cr^2+^:ZnSe/ZnS CPA: (**a**) input (red) and output (blue) idler spectra; (**b**) input (red) and output (blue) pump spectra; (**c**) input chirped pump (blue) and idler (red) pulse shapes; (**d**) calculated transform-limited pulse duration (FWHM) of the amplified idler: 18.0 fs.

The results from simulating the amplification of 4–12 *μ*m idler pulses in DC-OPA are shown in Fig. 7. The high-energy 2.3–2.5 *μ*m pump pulse can be generated directly in a multi-pass Cr^2+^:ZnSe/ZnS CPA by using a high energy (15 *μ*J) seed pulse generated in BIBO DFG. Thus, a regenerative amplifier can be avoided, which would make the synchronization between the pump pulse and the signal pulse rather difficult. The pump pulse is negatively stretched to 10 ps, while the 4–12 *μ*m idler pulse is positively stretched to 6.3 ps, as shown in Fig. 7(c). The input pump-to-idler energy ratio is assumed to be 10^4^. The input idler and pump intensities are 4.1 MW/cm^2^ and 20 GW/cm^2^, respectively. The ZGP thickness is set to be 0.76 mm with a Type I phase-matching angle of 48.5°. As shown in Fig. 7(a), the amplified idler spectrum spans from 4 *μ*m to 12 *μ*m, supporting 18.0 fs transform-limited sub-cycle pulses, as can be seen from Fig. 7(d). The pump-to-idler conversion efficiency reaches 27.1% (gain: 2706). The broad phase matching can be tailored for the amplification of the 4–12 *μ*m idler in ZGP to accommodate both a flexible pump bandwidth and a flexible center wavelength from any specific Cr^2+^:ZnSe/ZnS CPA laser.

**Figure 8:**
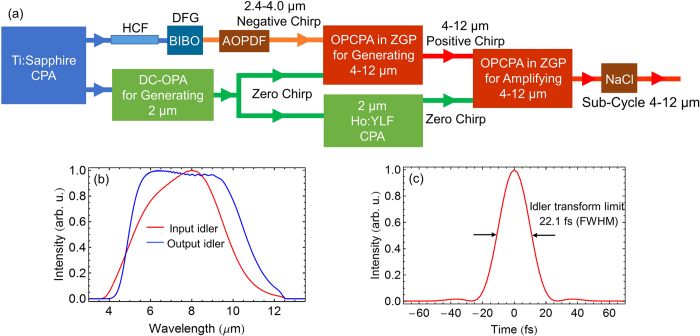
(**a**) Proof-of-principle schematic setup for amplifying 4–12 *μ*m idler pulses by OPCPA in a 0.7-mm ZGP pumped by a 2 *μ*m Ho:YLF laser; simulation results: (**b**) input (red) and output (blue) idler spectra & (**c**) calculated transform-limited pulse duration (FWHM) of the output idler: 22.1 fs.

For the sake of comparison, we also simulate the amplification of 4–12 *μ*m idler pulses by an OPCPA in ZGP pumped by a 2 *μ*m picosecond Ho:YLF laser. Recently, tremendous progress has been made on 2 *μ*m Ho:YLF/YAG CPA lasers^26^,^39^,^40^,^41^, among which 55 mJ, 4.3 ps, 1 kHz Ho:YLF laser system has been achieved. Subsequently, an OPA in ZGP pumped by a Ho:YAG CPA laser has generated 100 *μ*J, 69 fs (calculated transform limit) laser pulses at 5.3 *μ*m^26^, and an OPCPA in ZGP pumped by a Ho:YLF CPA laser has generated 200 *μ*J, 360 fs laser pulses at 7 *μ*m^25^. The MIR pulses in both cases have not been compressed to the near transform limit due to lack of higher-order phase compensation. The approach we proposed in Fig. 8(a) can not only generate and amplify a broader bandwidth (4–12 *μ*m) but also compress such broadband pulses to the near transform limit. The high-energy 2 *μ*m from the DC-OPA can seed directly a multipass Ho:YLF CPA, avoiding a regenerative amplifier that would complicate the synchronization between the pump and the idler in the 4–12 *μ*m OPCPA. The simulation results are shown in Fig. 8(b,c). The input pump centered at 2 *μ*m has a pulse duration (FWHM, transform-limited) of 10 ps and a peak intensity of 20 GW/cm^2^. The input idler pulses are stretched to 6.3 ps from 4 *μ*m to 12 *μ*m with a peak intensity of 4.4 MW/cm^2^. The input pump-to-idler energy ratio is 10^4^. The type I phase matching angle of ZGP is 53°. It can be seen from Fig. 8 that the amplified idler spectrum spans from 4 *μ*m to 12 *μ*m. The conversion efficiency from the pump to the idler is 13.2% (gain: 1317), which is much lower compared with that from the 4–12 *μ*m DC-OPA due to poorer phase matching and unfavorable pump-to-idler photon ratio. The compression of 4–12 *μ*m can also be achieved by indirect pulse shaping in the idler pulse generation process. The phase of the generated positively chirped 4–12 *μ*m pulse can be coarsely controlled by a NaCl crystal and finely controlled indirectly by controlling the phase of the 2.4–4.0 *μ*m signal pulse using a commercial AOPDF.

## Discussion

In an OPA, the parametric gain is defined as ref. 42:


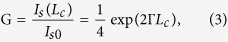


which grows exponentially with the crystal length *L*_*c*_ and nonlinear coefficient Γ, given as


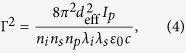


where *d*_eff_ is the effective nonlinearity, *I*_*p*_ is the pump intensity, *n*_*i,s,p*_ are the refractive indices of the idler, signal, and pump wavelengths respectively, *λ*_*i,s*_ are the wavelengths of the idler and signal, respectively, *ε*_0_ is the vacuum permittivity, and *c* is the speed of light in vacuum. The parametric gain, and thus the conversion efficiency, both grow exponentially with the crystal length and the square root of intensities. The maximum intensity allowed is limited by the crystal damage threshold, while the maximum length of crystal is limited by the preferred parametric gain bandwidth.

It would be meaningful to show quantitatively the conversion efficiencies with different pump intensities and crystal lengths, from which the energy scaling of specifically designed DC-OPA systems can be determined. For the OPCPA generating 4–12 *μ*m idler pulses discussed earlier, it is critical to maintain the 4–12 *μ*m broad gain bandwidth by using a ZGP crystal length shorter than 0.6 mm. Thus, we do not discuss the energy scaling of the first-stage OPCPA that is used to produce seed idler pulses since the energy scaling is determined by later stages of DC-OPAs. Here we focus on the energy scaling of the DC-OPA for amplifying 4–12 *μ*m idler pulses. Table 1 shows 15 instances: 5 pump-to-idler input energy ratios and 3 different input pump intensities for each different ratio. For each combination of pump-to-idler ratio and input pump intensity, we show the corresponding crystal lengths that were optimized to achieve optimum conversion efficiencies, and the corresponding transform limited pulse durations of the output idler pulses. It can be seen that, at a fixed input pump intensity, the conversion efficiency decreases only slightly and the pulse duration of idler pulses does not increase much when the pump-to-idler input ratio increases by four orders of magnitude. Meanwhile, at a fixed input energy ratio, the changes of conversion efficiency and the pulse duration of idler pulses are not obvious when the pump intensity changes from 20 GW/cm^2^ to 1.25 GW/cm^2^. Thus, both the conversion efficiency and the idler pulse duration (or parametric gain bandwidth) depend weakly on the crystal length varying from 0.45 mm to 4.4 mm, which is due to the excellent phase matching in the DC-OPA. Energy scaling can be obtained by using Table 1. For example, at 10 ps pump pulse duration and 20 GW/cm^2^ intensity, a commercially available 20-mm-diameter ZGP crystal can withstand a pulse energy of 600 mJ. If the input idler energy is assumed to be around 1 *μ*J, the output idler energy would be 157 mJ with a conversion efficiency of 26.2%, which gives sub 10 TW peak power with a sub-cycle pulse duration of 18.5 fs.

Table 2 shows quantitatively the crystal lengths, idler pulse durations and conversion efficiencies for 5 pump-to-idler input energy ratios and 3 different input pump intensities for each different ratio in the OPCPA pumped by the Ho:YLF CPA laser. It can be seen that, at a fixed input pump intensity, the conversion efficiency decreases considerably and the pulse duration of idler pulses increases significantly when the pump-to-idler input idler ratio increases by four orders of magnitude. Meanwhile, at a fixed input energy ratio, the conversion efficiency also decreases substantially and the pulse duration of idler pulses increases considerably when the pump intensity decreases from 20 GW/cm^2^ to 1.25 GW/cm^2^. The decrease in conversion efficiency and increase in pulse duration is due to the use of a longer crystal, which inevitably results in poorer phase matching: 65.5 fs, 4.0% in a 4 mm ZGP crystal versus 20.1 fs, 14.1% in a 0.37 mm one. Thus, it is much more favorable to use a short ZGP crystal in the 4–12 *μ*m OPCPA pumped by a 2 *μ*m pump source, which can be achieved by using a short pump pulse at a few picosecond and a high-energy input idler around or above *μ*J level. Suppose the input idler energy is around 1 *μ*J, the output idler energy would be 71 mJ with a 600 mJ pump, and the pump-to-idler conversion efficiency would be 11.9%, giving 2.9 TW peak power with a 24.4 fs (FWHM) pulse duration.

Several critical issues should be discussed here. First, it should be noticed that a single crystal was used in the simulation for amplification of idler pulses. In the actual experimental setup, two stages might be needed for achieving tens of mJ idler energy, where the first stage provides high gain with a relatively low efficiency and a second stage is used to amplify the idler efficiently. Second, the B-integral of a 157 mJ 4–12 *μ*m pulse in a 1-mm thick ZPG with 10 GW/cm^2^ intensity is estimated to be around 0.3 (n_2_ = 400 × 10^−16^ cm^2^/W^43^), which is acceptable. Third, the superfluorescence buildup would be small because of high seed energy (several *μ*J). In addition, in order to preserve the passive CEP stability of the idler pulses in the generation stage, it is critical to maintain the time delay between the pump pulses and the signal pulses, which can be achieved by interferometric locking of the optical path length difference between the pump and the idler beams (20 as RMS)^44^.

## Conclusions

By tailoring the phase matching of the nonlinear crystal ZGP, we propose to generate 4–12 *μ*m idler pulses in OPCPA pumped by a narrowband 2 *μ*m laser and then amplify the idler pulses in DC-OPA pumped by a Cr^2+^:ZnSe/ZnS CPA centered around 2.4 *μ*m. The 4–12 *μ*m pulses can be compressed to sub-cycle pulses by controlling the signal (2.4–4.0 *μ*m) phase using a commercially available AOPDF combined with a bulk material NaCl. The parametric gain bandwidth and conversion efficiency from the 4–12 *μ*m DC-OPA are barely limited by the ZGP crystal length, which can support up to 10 TW peak power, provided that the ZGP crystal can withstand 20 GW/cm^2^ peak intensity of the Cr^2+^:ZnSe/ZnS pump laser. Similarly, we propose to amplify high-energy 4–12 *μ*m sub-cycle pulses via an OPCPA pumped by a narrowband 2 *μ*m Ho:YLF laser. Unlike the 4–12 *μ*m DC-OPA, the OPCPA is significantly limited by the ZGP crystal length-both the parametric gain bandwidth and conversion efficiency. However, such an OPCPA can still support up to 3 TW peak power.

## Additional Information

**How to cite this article**: Yin, Y. *et al*. Towards Terawatt Sub-Cycle Long-Wave Infrared Pulses via Chirped Optical Parametric Amplification and Indirect Pulse Shaping. *Sci. Rep.* **7**, 45794; doi: 10.1038/srep45794 (2017).

**Publisher's note:** Springer Nature remains neutral with regard to jurisdictional claims in published maps and institutional affiliations.

## Figures and Tables

**Table 1:** Simulation of DC-OPAs in ZGP pumped by a 2.4 *μ*m Cr^2+:^ZnSe/ZnS laser.

Pump-to-idler energy ratio	ZGP length (mm)			Idler transform limit (fs)			Conversion efficiency		
	Input pump (GW/cm^2^)			Input pump (GW/cm^2^)			Input pump (GW/cm^2^)		
	20.0	5.00	1.25	20.0	5.00	1.25	20.0	5.00	1.25
10^6^	1.10	2.20	4.40	18.5	18.8	19.9	26.2%	26.1%	25.4%
10^5^	0.94	1.88	3.76	18.0	18.3	19.1	26.6%	26.4%	25.8%
10^4^	0.76	1.52	3.04	18.0	18.2	18.8	27.1%	26.8%	25.9%
10^3^	0.60	1.20	2.40	17.3	17.7	18.2	27.7%	27.4%	26.6%
10^2^	0.45	0.90	1.80	16.8	16.9	17.4	29.1%	28.8%	28.0%

**Table 2:** Simulation of OPCPAs in ZGP pumped by a 2 *μ*m Ho:YLF laser.

Pump-to-idler energy ratio	ZGP length (mm)			Idler transform limit (fs)			Conversion efficiency		
	Input pump (GW/cm^2^)			Input pump (GW/cm^2^)			Input pump (GW/cm^2^)		
	20.0	5.00	1.25	20.0	5.00	1.25	20.0	5.00	1.25
10^6^	1.00	2.00	4.00	24.4	32.1	65.5	11.9%	8.0%	4.0%
10^5^	0.85	1.70	3.40	23.3	29.8	56.6	12.6%	8.8%	4.4%
10^4^	0.70	1.40	2.80	22.1	27.6	45.8	13.2%	9.6%	4.8%
10^3^	0.55	1.10	2.20	20.9	24.7	34.1	13.7%	10.3%	5.3%
10^2^	0.37	0.74	1.48	20.1	21.9	24.9	14.1%	11.1%	6.2%
